# Characterization of the Primary Metabolome of *Brachystegia boehmii* and *Colophospermum mopane* under Different Fire Regimes in Miombo and Mopane African Woodlands

**DOI:** 10.3389/fpls.2017.02130

**Published:** 2017-12-14

**Authors:** Jossias A. Duvane, Tiago F. Jorge, Ivete Maquia, Natasha Ribeiro, Ana I. F. Ribeiro-Barros, Carla António

**Affiliations:** ^1^Faculty of Sciences, Eduardo Mondlane University, Maputo, Mozambique; ^2^Plant Metabolomics Laboratory, Instituto de Tecnologia Química e Biológica António Xavier, Universidade Nova de Lisboa, Oeiras, Portugal; ^3^Biotechnology Center, Maputo, Mozambique; ^4^Faculty of Agronomy and Forest Engineering, Eduardo Mondlane University, Maputo, Mozambique; ^5^Plant Stress and Biodiversity Laboratory, Linking Landscape, Environment, Agriculture and Food, Instituto Superior de Agronomia, Universidade de Lisboa, Lisboa, Portugal

**Keywords:** tree legumes, Miombo ecosystem, Mopane ecosystem, GC-TOF-MS, primary metabolome, fire tolerance

## Abstract

Miombo and Mopane are ecological and economic important woodlands from Africa, highly affected by a combination of climate change factors, and anthropogenic fires. Although most species of these ecosystems are fire tolerant, the mechanisms that lead to adaptive responses (metabolic reconfiguration) are unknown. In this context, the aim of this study was to characterize the primary metabolite composition of typical legume trees from these ecosystems, namely, *Brachystegia boehmii* (Miombo) and *Colophospermum mopane* (Mopane) subjected to different fire regimes. Fresh leaves from each species were collected in management units and landscapes across varied fire frequencies in the Niassa National Reserve (NNR) and Limpopo National Park (LNP) in Mozambique. Primary metabolites were extracted and analyzed with a well-established gas chromatography time-of-flight mass spectrometry metabolomics platform (GC-TOF-MS). In *B. boehmii*, 39 primary metabolites were identified from which seven amino acids, two organic acids and two sugars increased significantly, whereas in *C. mopane*, 41 primary metabolites were identified from which eight amino acids, one sugar and two organic acids significantly increased with increasing fire frequency. The observed changes in the pool of metabolites of *C. mopane* might be related to high glycolytic and tricarboxylic acid (TCA) rate, which provided increased levels of amino acids and energy yield. In *B. boehmii*, the high levels of amino acids might be due to inhibition of protein biosynthesis. The osmoprotectant and reactive oxygen species (ROS) scavenging properties of accumulated metabolites in parallel with a high-energy yield might support plants survival under fire stress.

## Introduction

The tropical forest and savannas constitute one sixth of global terrestrial surface and more than a half of the African continent. The vegetation portions of these ecosystems possess about 53.000 plant species and correspond to 30% of global primary production. These ecosystems have a high ecological and socio-economic impact being extremely important (i) in maintaining water, carbon and energy balance, (ii) as a source of high-quality wood, and (iii) and being highly used to treat animal and human diseases in local traditional medicine (White, 1983; Campbell et al., 1996; Ribeiro et al., 2010, 2012). The woodlands from southern Africa are composed mainly by strands of Miombo and Mopane (Allen et al., 2002; Bond et al., 2005; Neary et al., 2008). Miombo is dominated by the genera *Brachystegia*, *Julbernardia*, and *Isoberlinia*, while Mopane is nearly monospecific with the predominance of *Colophospermum mopane*. Miombo and Mopane ecosystems are frequently exposed to animal and human action and to adverse abiotic conditions, such as high temperature, ultraviolet irradiation, and drought. These factors are intimately associated with the occurrence of fires, whose regime can affect the structure and composition of the vegetation, thereby threatening the ecosystem’s stability (Bohnert and Shen, 1998; Apel and Hirt, 2004; Shulaev et al., 2008).

Although much of the terrestrial vegetation is frequently affected by fire, the analysis of its impact in biological systems is poorly understood (Neary et al., 2008). Fire occurs at different frequencies and intensities that can result in a variety of ecological effects. When controlled, fire promotes dense and rapid regrowth, accelerating the natural cycle of primary production and respiration (Allen et al., 2002; Bond et al., 2005). In contrast, when not controlled, fire is associated with the increase of the superficial temperature and smoke concentration the reduction of humidity and water permeability due to the accumulation of hydrophobic elements in the soil, which in the last instance, determines seasonality distribution of nutrients (Solomon et al., 1994; Ledgards and Steelek, 2016). In addition, the morphology and physiology of *B. boehmii* and *C. mopane* are severely affected, e.g., aerial components of shrubs are destroyed and plant height and stem circumference are reduced (Henning, 1976, unpublished; Kennedy and Potgieter, 2003; Gandiwa and Kativu, 2009).

To cope with fire stress, *B. boehmii* and *C. mopane* have evolved morphology and physiology strategies. The roots and leaves of these plants are crucial organs that respond to changes in the surrounding environment that enables their survival under these adverse environmental conditions. These plants have high fin root densities (i.e., 20–120 cm depth) to facilitate quick water and nutrient uptake and transport (Smit and Rethman, 1998; Mantlana, 2002), and also have the ability to produce root suckers, which allow the shoots to grow faster than newly established seedlings (Luoga et al., 2004). Their leaves have leathery membranes that act as a buffer layer to avoid the heat and help reducing the rate of water loss through evapotranspiration. In addition, these leaves have a tendency to fold together, especially when the temperature exceeds 30°C (Madams, 1990).

*Colophospermum mopane*, in particular, contains crystals of calcium oxalate, which enhance wood resistance to fire through the production of a high CO_2_ concentration; a high concentration of CO_2_ acts as fire retardant by reducing the flame length (CICA, 1996). The resulting smoke is also composed by a diversity of volatile compounds, such as O_3_, SO_2_, and NO_2_, which promote the destruction of chlorophyll, inhibit the K^+^ channels that regulate guard cell functions (Shimazaki et al., 1984; Wellburn, 1985; Torsethaugen et al., 1999), reduce stomatal conductance, inhibit photosynthetic O_2_ evolution to the electron transport system, inactivate Calvin-Cycle enzymes, and reduce the main plant antioxidant enzymes (i.e., superoxide dismutase and glutathione reductase) (Tanaka et al., 2014).

Like other abiotic stresses, fire may cause osmotic stress that result in the increase of intracellular levels of reactive oxygen species (ROS). This process is characterized by dehydration and ion accumulation in the cytoplasm, inducing oxidative damage of proteins, nucleic acids, and lipids, leading to a loss of metabolic homeostasis (Bohnert and Shen, 1998; Apel and Hirt, 2004).

Some plant species tolerate environmental stresses through the activation of complex adaptative mechanisms. These include stress detection and activation of signal transduction mechanisms that ultimately lead to the production of antioxidants and accumulation of compatible solutes (e.g., sugars, amino acids, some amino acid derivatives, polyols, fructans and quaternary amino and sulfonium compounds) to acquire the new homeostatic state (Shulaev et al., 2008). Antioxidants prevent oxidative damage by “scavenging” ROS while compatible solutes alleviate the inhibitory effect of high ion concentration on enzyme activity, stabilize proteins and membranes, and also act as ROS “scavengers” (Solomon et al., 1994; Ledgards and Steelek, 2016).

Miombo and Mopane species, including *B. boehmii* and *C. mopane*, are widely reported to be fire tolerant and to have distinct reproductive and survival strategies in relation to fire, which might function as a potent biological filter (Stalmans et al., 2004; Shulaev et al., 2008). However, the metabolic mechanisms of resistance to this abiotic factor are unknown. Besides, there is few and incipient information regarding the primary metabolite composition of the species from both ecosystems (Chagonda et al., 1999; Ferreira et al., 2003) and, therefore, the novelty and pioneering aspect of this work.

Plants have high metabolite diversity with distinct physico-chemical properties and multiple analytical platforms are often used for metabolite analysis, particularly those based on nuclear magnetic resonance (NMR) and mass spectrometry (MS). Because of its higher sensibility, MS methodologies are by far more used in metabolomics studies than NMR methodologies (Fernie et al., 2004); however, no single analytical platform is capable to analyze simultaneously the total metabolite pool present in complex mixtures, and therefore, metabolomics studies based in MS typically combine the use of complementary separation techniques, such as gas chromatography (GC) and liquid chromatography (LC) (Fernie et al., 2004; Jorge et al., 2016). In this study, a well-established gas chromatography coupled to time-of-flight mass spectrometry (GC-TOF-MS) platform was used to characterize the primary metabolome of *B. boehmii* and *C. mopane*, two woody leguminous species from the Miombo and Mopane vegetation, subjected to different fire regimes. Upon increasing fire frequency, a prominent variation of some primary metabolites was observed, which might reflect their antioxidant and osmoprotectant roles in adaptive mechanism responses of these species to fire.

## Materials and Methods

### Study Area and Sampling

**FIGURE 1:**
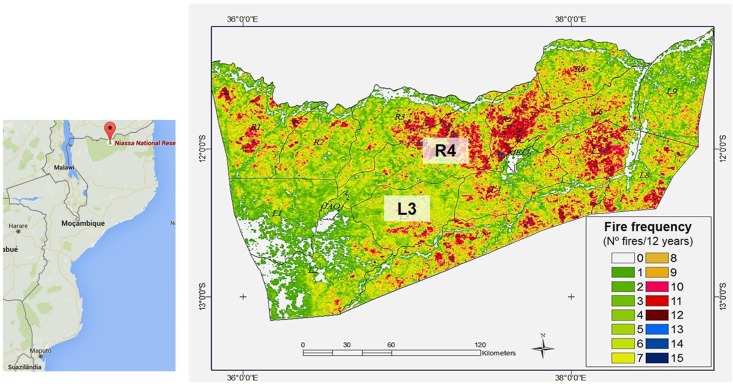
Niassa National Reserve (NNR) fire areas map: L3 (LFF) and R4 (HFF) corresponds to *B. boehmii* sampling areas (Cangela, 2014).

The study areas were located in the Niassa National Reserve (NNR) and Limpopo National Park (LNP). NNR is the most intact part of Miombo vegetation and represents one of the least disturbed areas of Africa’s deciduous miombo woodlands, with a superficial extension of 42.000 Km^2^. It is located in the Niassa Province, north of Mozambique, and it is limited in the north by Tanzania, and in the east and south by the Lugenda river (WWF, 2014). This reserve is characterized by humid tropical climate with an average annual rainfall between 600 and 1400 mm during the months of April and November, hottest average temperature of 30°C, low fertile sandy-clayey soils, and according to data from MODerate Resolution Imaging Spectroradiometer (MODIS) sensor on board of NASA’s Terra and Aqua Satellites, the burnt area in NNR corresponded to annual to tri-annual fires (SRN, 2005; Ribeiro et al., 2012). For management purposes, the reserve was divided in several management units (MUs), namely, R1–R6 and L1–L9. In this study, R4 and L3 were selected according to geographic proximity and differences in fire frequency: R4 with high fire frequency (HFF) (10–15 times in 12 years; the last fire took place 8 months before sampling) and L3 with low fire frequency (LFF) (3–5 times in 12 years; the last fire took place 7 months before sampling) (Cangela, 2014) (**Figure 1** and Supplementary Table S1).

Limpopo National Park is part of the Mopane vegetation and constitutes the second most important vegetation area, with 10.000 Km^2^ of superficial extension, located in the southern part of Gaza Province, south of Mozambique, on the eastern boundary of the Kruger National Park (Stalmans et al., 2004; MAE, 2005). LNP is characterized by subtropical climate with an average annual rainfall between 360 and 500 mm during the months of March and August, hottest average temperature of 40°C, limestone and rocky soils, and according to data from MODIS sensor on board of NASA’s Terra and Aqua Satellites, the burnt area in LPN showed a quasi-biannual pattern, with highest burnt areas occurring in 2004, 2006, and 2008 (MAE, 2005; Govender et al., 2015).

**FIGURE 2:**
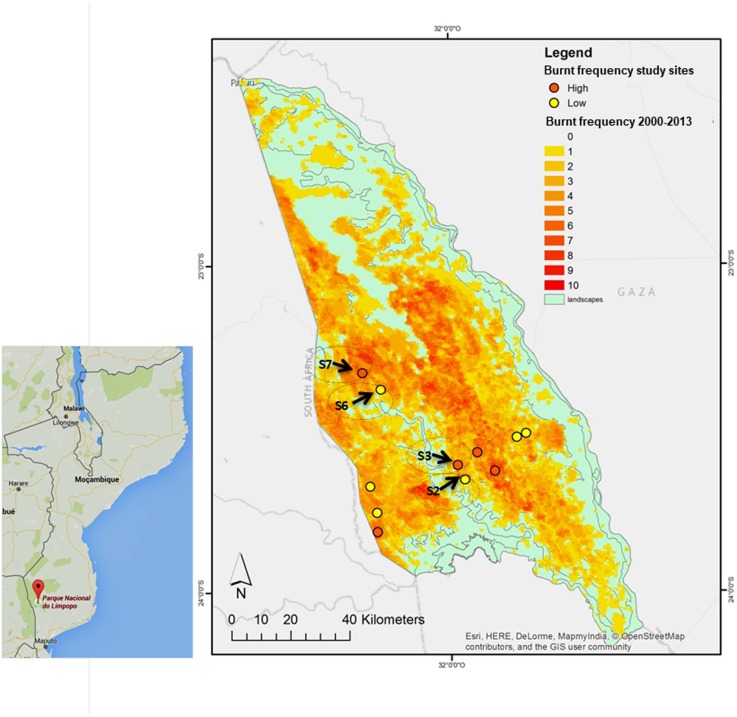
Limpopo National Reserve (LNR) fire areas map: S2, S6 (LFF) S3, S7 (HFF) corresponds to *C. mopane* sampling areas (Navashni et al., 2015).

The park was divided in landscapes, namely, S1–S12. In this study, S2, S3 from Calcrete area, and S6, S7 from Lebombo north area, were selected according to geographic location and differences in fire frequency: S2 and S6 with LFF (2–3 times in 10 years; in S2, the last fire took place 10 months before sampling whereas in S6 the last fire took place 33 months before sampling), S3 and S7 with HFF (8–10 times in 10 years; the last fire took place 8 months before sampling and 20 months before sampling, respectively). All of them have burnt at least twice between 2010 and 2013 (Govender et al., 2015) (**Figure 2** and Supplementary Table S2).

Due to the dynamics of Miombo and Mopane ecosystems (**Figures 1**, **2**), areas without fire incidence are not available; actually, low fire frequencies are indeed the “positive controls” on the ecology of these ecosystems (Ribeiro et al., 2017). Samples were obtained using the Stratified Random sampling, where the population was first divided into strata according to the fire frequency. Then, in each site and MU, four linear transects (each with 160 m and four plots, distance between plots was 40 m) were delineated (replicas). In the center of each plot, six young undamaged leaves were collected per plant. However, due to the remoteness of the areas under study, fewer sites were accessed.

Samples of *B. boehmii* in NNR were collected between June and July 2012 (Supplementary Table S1) where average temperature of sampling sites was 22°C and rainfall 1.5 mm. Samples of *C. mopane* in LNP were collected in June 2014 (Supplementary Table S2), where the average temperature of collecting sites was 17.5°C and raifall 73.25 mm. For each sampling point, the environmental variables and fire events were recorded (Supplementary Tables S1, S2). Leaves were stored in silica gel rubin (Fluka) for about 1 week (the time of the trip from the field to the lab) and then frozen in liquid nitrogen and stored at -80°C at the Biotecnology Center Lab of the Eduardo Mondlane University until lyophilization and subsquent metabolite analysis.

### Primary Metabolite Extraction, Derivatization and GC-TOF-MS Analysis

Primary metabolites were extracted following a previously described protocol (Lisec et al., 2006). Briefly, lyophilized leaves of 20 *B. boehmii* plants and 40 *C. mopane* plants were homogenized separately using a mortar and pestle filled with liquid nitrogen. Approximately 20 mg (DW, dry weight) were weighed into 2.0 mL safe-lock polypropylene microfuge tubes (Eppendorf, Hamburg, Germany) and primary metabolites were extracted using 1400 μL of methanol (100%) containing ribitol (0.2 mg mL^-1^ in water) as internal standard. The mixture was incubated on a shaker at 70°C for 15 min (Thermomixer C, Eppendorf, Hamburg, Germany) and centrifuged for 10 min at 11000 ×*g* (Centrifuge 5430, Eppendorf, Hamburg, Germany) at 25°C. The supernatant was transferred to a glass vial (Schott GL14, Mainz, Germany) and 750 μL of chloroform were added followed by 375 μL of distilled water. The resulting mixture was vortex-mixed for 15 s, and centrifuged for 15 min at 2200 ×*g* (Centrifuge 5810R, Eppendorf, Hamburg, Germany). Finally, 150 μL of the upper phase (polar phase) of each sample were transferred to a new 1.5 mL microfuge tube (Eppendorf, Hamburg, Germany) and dried down with a vacuum concentrator (Concentrator CentriVap, Labconco, Kansas City, MO, United States) for 3 h. Biological variations were controlled by analyzing quality control (QC) standards by fatty acid methyl esters (FAMES) internal standard markers and a QC standard solution of 41 pure reference compounds (i.e., the most detected and abundant metabolites) throughout the analysis. The final dried extracts were filled with argon gas to avoid oxidation and degradation of extracted metabolites by reactions with atmospheric air components, and stored at -80°C prior to derivatization and GC-TOF-MS analysis. Extracted primary metabolites were derivatized and analyzed with gas chromatography coupled to time-of-flight mass spectrometry (GC-TOF-MS) according to a well-established GC-TOF-MS protocol (Lisec et al., 2006). The system consisted of a gas chromatograph (6890N Plus, Agilent Technologies, Böblingen, Germany) coupled to a TOF-MS (Pegasus III TOF MS system, LECO, Mönchengladbach, Germany), in which 1 μL of sample were injected (Supplementary Files S1, S2).

### Data Analysis and Statistics

After GC-TOF-MS analysis, the obtained files (cdf. format) with each chromatogram and mass spectra for each sample were analyzed using AMDIS (Automated Mass Spectral Deconvolution and Identification System) software (v 2.71). Primary metabolites were annotated using the TagFinder software, matching mass spectral and retention time index to the reference collection of authenticated standard substances from the Golm Metabolome Database (Luedemann et al., 2008). The relative abundance of primary metabolites was normalized dividing the corresponding values by the dry weight (DW) of the samples and the signal intensity of the internal standard (ribitol) (Supplementary Files S1, S2).

Significant differences between LFF and HFF samples were estimated with Student *t*-test (*p* < 0.05) using the R-software (R Core Team, 2016) (Supplementary Tables S1–S5). The *p*-values were corrected for multiple comparisons using the Benjamini–Hochberg False Discovery Rate (FDR) correction (Supplementary File S3). Principle Component Analysis (PCA), Partial Least Squares Discriminant Analysis (PLS-DA) and Heatmap plots were obtained in the R software using the packages: pcamethods (Stacklies et al., 2007), mixOmics (Le Cao et al., 2016) and pheatmap (Raivo, 2015).

## Results

### Primary Metabolite Profiling of *B. boehmii*

**FIGURE 3:**
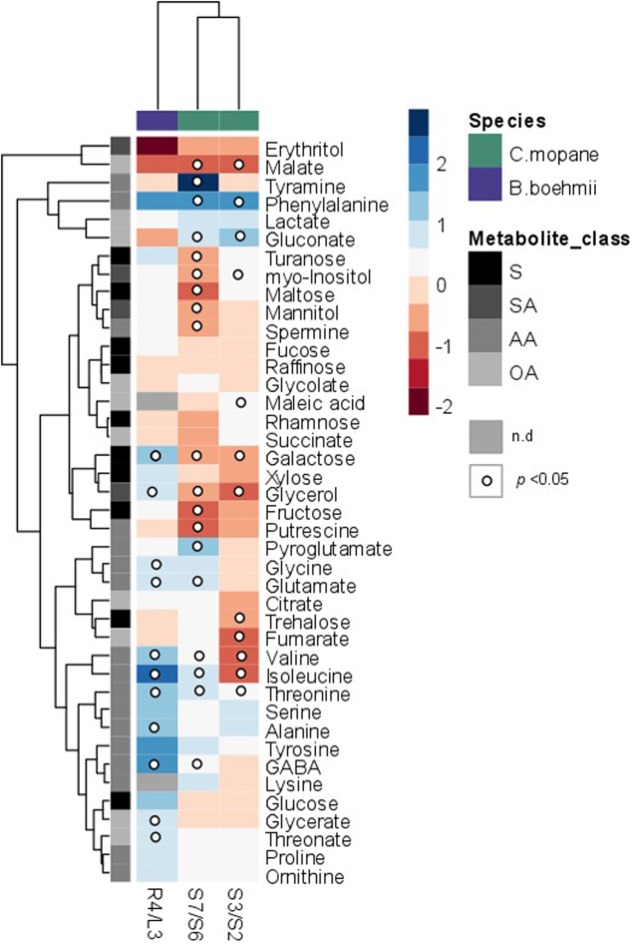
Heatmap showing metabolite responses in *C. mopane* and *B. boehmii* under different fire regimes. Metabolites were determined as described in Section “Material and Methods.” Relative values are normalized to the internal standard (ribitol) and dry weight (DW) of the samples. Values presented as means ± SE of three to five independent measurements. Dots indicate that the differences are statistically significant by Student’s *t*-test (*p* < 0.05). Gray-color squares represent not detected (n.d) values. False-color imaging log2-transformed GC-TOF-MS data. AA, amino acids; OA, organic acids; S, sugars; SA, sugar alcohols.

**FIGURE 4:**
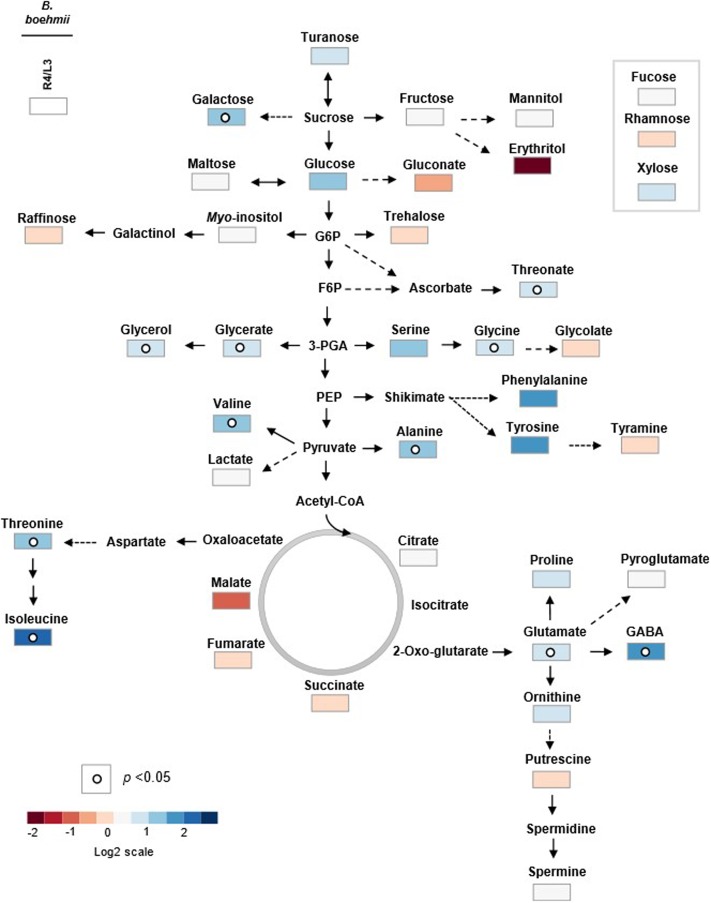
Primary metabolic pathway reflecting the metabolite changes in *B. boehmii* under low fire frequency (L3) and high fire frequency (R4). The relative values are represented by log2 of the ratio between R4/L3 ± SE of ten accessions. Relative values were normalized to the internal standard (ribitol) and dry weight (DW) of the samples. Dots indicate that the differences are statistically significant by Student’s *t*-test (*p* < 0.05).

GC-TOF-MS analysis allowed the identification of 39 primary metabolites in *B. boehmii*, namely, 14 amino acids, ten sugars, nine organic acids, four polyols, and two polyamines, whose relative abundance varied prominently between high fire frequency (HFF) and low fire frequency (LFF) samples (**Figures 3**, **4** and Supplementary Table S3). Increasing fire frequency caused a significant (*p* < 0.05) increase of the aminoacids glycine (Gly), alanine (Ala), valine (Val), glutamate (Glu), γ-amino butyric acid (GABA), isoleucine (IIe) and threonine (Thr). In respect to sugars, a significant increase was observed for galactose (Gal) with increasing fire frequency, but in general, most sugars remained unchanged. The sugar alcohol glycerol was observed to significantly (*p* < 0.05) increase with increasing fire frequency. Just over a third of organic acids significantly increased with increasing fire frequency, namely threonate and glycerate.

Principle component analysis analysis allowed for the identification of patterns between the LFF and the HFF *B. boehmii* samples. The first two principal components (PC1 and PC2) accounted for 57.09% of the total variance and revealed a separation between the two group samples (**Figure 6A**). To gain further insights on the fire-responsive metabolites that discriminate as much as possible the group samples, we have then applied a validated supervised PLS-DA model (**Figure 7A**). Our PLS-DA models were validated based on the criterion of lowest classification error rate using the leave-one-out cross-validation embedded in the R package “mixOmics” (Supplementary File S4). PLS-DA analysis revealed to be effective in separating the LLF samples from the HFF samples (**Figure 7A**) and we were able to identify that those metabolites that significantly increased (*p* < 0.05) in HFF were also responsible for the clustered of these group of samples (**Figure 7A**).

An author metabolite pathway map of *B. boehmii* (**Figure 4**) was generated in order to predict sites of metabolic regulation. The metabolite pathway map shows that HFF results in the accumulation of many sugars, which may reveal low glycolysis ratio, and low amino acids precursors from glycolysis (glucose-6-phosphate (Glc-6-P) and pyruvate (Pyr)) and TCA [oxaloacetate (OAA) and 2-oxo-glutarate (2OG)], however, the vast majority of amino acids accumulated, which may be due to other factors aside from central metabolism.

### Primary Metabolite Profiling of *C. mopane*

**FIGURE 5:**
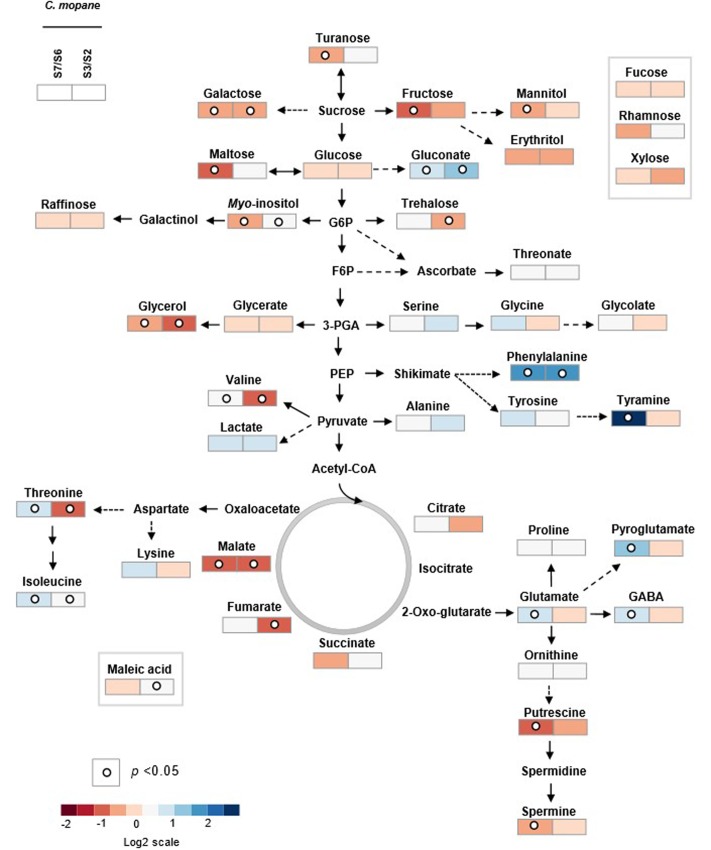
Primary metabolic pathway reflecting the metabolite changes in *C. mopane* under different fire frequency regimes, low fire frequency (S6 and S2) and high fire frequency (S7 and S3). The relative values are represented by log2 of the ratio between S7/S6 ± SE and of the ratio between S3/S2 ± SE of ten accessions. Relative values are normalized to the internal standard (ribitol) and dry weight (DW) of the samples. Dots indicate that the differences are statistically significant by Student’s *t*-test (*p* < 0.05).

In *C. mopane* 41 primary metabolites were identified, namely 15 amino acids, ten sugars, ten organic acids, four polyols and two polyamines (**Figures 3**, **5** and Supplementary Tables S4, S5), whose relative abundance varied notably between HFF and LFF samples. Comparing the metabolite profiles of trees from Lebombo north landscapes S6-LFF *versus* S7-HFF (**Figures 3**, **5** and Supplementary Table S4), the vast majority of amino acids levels increased significantly (*p* < 0.05) with the increasing of fire frequency, particularly Phe (up to threefold) and tyramine (up to sevenfold). On the other hand, HFF resulted in the decrease of the majority of sugars, but only fructose (Fru), Gal, maltose and turanose decreased significantly. More than a half of organic acids showed a tendancy to accumulate. Almost all sugar alcohols, namely, glycerol, mannitol and *myo*-inositol as well as the polyamines putrescine (Put) and spermine (Spm) were shown to decrease significantly.

For the metabolite profiles between trees from Calcrete landscapes S2-LFF and S3-HFF (**Figures 3**, **5** and Supplementary Table S5) only a couple of the amino acids significantly increased with increasing fire frequency, namely, Thr and Phe which accumulated up to threefold. On the other hand, only two amino acids, Ile and Val, decreased significantly (*p* < 0.05). Among sugars and sugar alchohols, the relative abundance of Gal, trehalose (Tre) and glycerol decreased significantly with increasing fire frequency whereas *myo*-inositol increased. Only a few organic acids increased significantly with the fire frequency, namely, malate and gluconate. On the other hand, the levels of the tricarboxilic acid (TCA)-cycle intermediates fumarate and malate decreased significantly.

**FIGURE 6:**
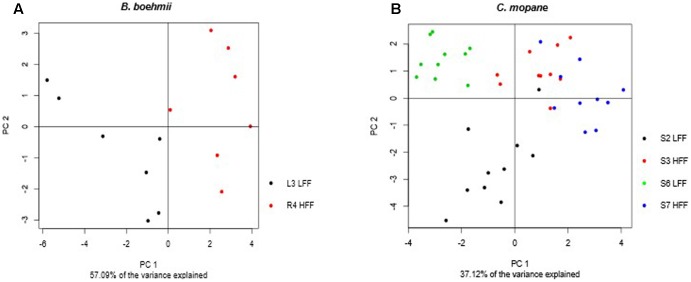
Principal component analysis (PCA) score plots of metabolic profiles for **(A)** *B. boehmii* under low fire frequency (L3) and high fire frequency (R4) and **(B)** *C. mopane* under different fire frequency regimes, low fire frequency (S6 and S2) and high fire frequency (S7 and S3).

**FIGURE 7:**
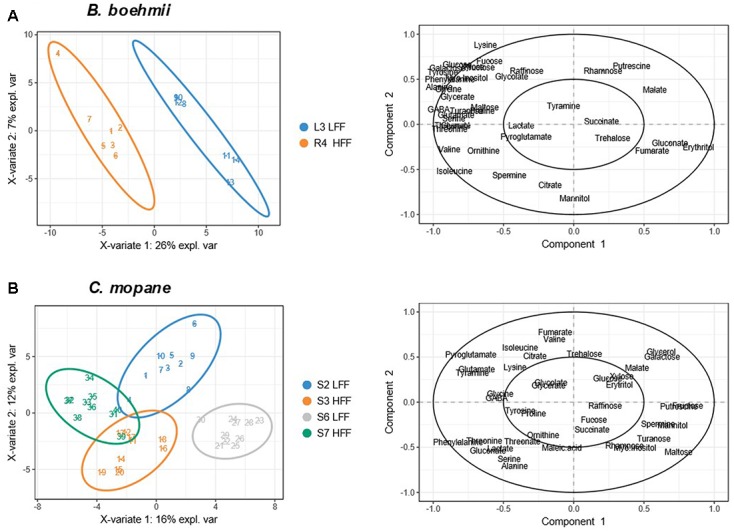
Partial Least Square Discrimination (PLS-DA) score and loading plots for **(A)** *B. boehmii* under low fire frequency (L3) and high fire frequency (R4) and **(B)** *C. mopane* under different fire frequency regimes, low fire frequency (S6 and S2) and high fire frequency (S7 and S3).

The PCA analysis of the *C. mopane* samples based on the first two principal components that accounted for 37.12% of the total variance also revealed a separation between the LFF and the HFF group samples (**Figure 6B**). In additon, the PLS-DA analysis revealed to be effective in separating the LLF samples of the S6 MU from the HFF of the S7 MU as well as the LFF samples of the S2 MU from the HFF of the S3 MU (**Figure 7B**). Moreover, the samples from the S6 MU were clearly separated from those of the different MUs. Regarding the loading plots, it was observed that while S6-LFF samples are separated from S7-HFF samples by the vertical axis (*y*-axis), the separation of S2-LFF samples from S3-HFF is visble by the horizontal axis (*x*-axis) (**Figure 7B**). Following this, the metabolites that significantly increased (*p* < 0.05) under HFF were clustered in the negative side of the *y*- and *x*-axis, respectively. On the other hand, the metabolites significantly decrease (*p* < 0.05) in the HFF samples were clustered in the positive side of the *y*- and *x*-axis.

To predict sites of metabolic regulation an author metabolite pathway map of *C. mopane* was generated (**Figure 5**). The map shows general decreasing of sugar concentration, which may show high glycolysis ratio, and probably related with the increasing of some TCA-cyle intermediate concentrations (malate, succinate in S2 and S3 landscapes, citrate, fumarate in S6 and S7 landscapes). In the context of HFF, it is also notable that some of glycolysis (Glc-6-P) and TCA (OAA and 2-OG) intermediates were probably the precursors of the biosynthesis of the vast majority of accumulated amino acids.

## Discussion

Environmental stresses lead to a loss of metabolic homeostasis in plants, which can be characterized by an imbalance between the production of ROS and antioxidant molecules (Mittler, 2006). The excess of ROS causes oxidative damage in biomolecules, such as, proteins, nucleic acids, and lipids (Manderscheid et al., 1992; Martinelli et al., 2007). According to our data, HFF increases the relative abundance of amino acids in both *B. boehmii* and *C. mopane* of the Lebombo north area and some amino acids of the Calcrete area. This profile can be associated with the adaptation of these species to stress caused by fire. Plants under abiotic stress accumulate amino acids in their cytoplasm, that play an important osmoprotectant role by helping plant cells to maintain their cellular turgor as well as to stabilize cellular membranes thereby preventing nucleic acid, protein, and lipid degradation (Mittler, 2006; Martinelli et al., 2007). Furthermore, amino acids can synergistically interact with phenols to form redox systems to “scavenge” ROS, enhancing the process of stress tolerance (Bolouri-Moghaddam et al., 2010). The accumulation of the non-protein amino acid GABA is probably related to signaling and regulation of different mechanisms of stress response, such as carbon and nitrogen balance, osmotic potential, ROS “scavenging” and pH regulation (Kinnersley and Turano, 2000; Lancien and Roberts, 2006). On the other hand, the accumulation of proteinogenic amino acids can reveal the inhibitory effect of heat in protein synthesis. Furthermore, the higher relative abundance of Val is probably linked to fire tolerance, as it has been reported to be strongly related to heat stress, but the specific mechanism is still unknown (Yu et al., 2012).

In respect to *B. boehmii*, the increase in fire frequency led to a general increase of molecules with osmoprotectant properties, such as sugars, and a decrease of TCA-cycle intermediates, which suggests the occurrence of low glycolysis and energy production rates, namely, ATP, FADH_2_ and NADH (Dias et al., 2015). Like amino acids, sugars can act as osmolytes, that contribute to maintain the cellular turgor, membrane stabilization and prevent protein degradation (Dias et al., 2015). The opposite was observed in *C. mopane*, except for maltose and *myo*-inositol. The decrease in the relative abundance of sugars was more prominent in trees from Libombo north than from Calcrete, but in both cases, were inversely related to the relative abundances of TCA-cycle intermediates, suggesting a parallel increase of the glycolysis and energy production rates (e.g., ATP, FADH_2_ and NADH) with increasing fire frequency (Dias et al., 2015). The higher amino acid levels can be a reaction to fire stress and similar profiles were observed in *Acacia ampliceps Maslin* exposed to salt stress (Sanchez et al., 2008; Theerawitaya et al., 2015), suggesting that the osmoprotective role of amino acids is the common response of many plant species to abiotic stress.

Polyols and organic acids are another type of molecules involved in osmoprotection and maintenance of metabolic processes under abiotic stress (Sanchez et al., 2008; Xu et al., 2008; Theerawitaya et al., 2015). The accumulation of glycerol in *B. boehmii* and *myo*-inositol in *C. mopane* might be related to the scavenging of hydroxyl radicals (Xu et al., 2008) while the accumulation of threonine might be linked to the maintenance of ionic balance at the membrane level (Bolouri-Moghaddam et al., 2010).

The changes in the pool of TCA-cycle intermediates along with the decrease of sugars in *C. mopane* might be related with an increased rate of glycolysis for use in the TCA cycle, which in turn provides precursors for other reactions such as the biosynthesis of amino and organic acids. In addition, it also provides chemical energy in the form of adenosine triphosphate (ATP) and nicotinamide adenine dinucleotide hydrogen (NADH) which might be needed to support plant survival during fire stress. The opposite behavior was observed in *B. boehmii*, where the decreased pool of TCA-cycle intermediates agreed with an increase of several sugars; this fact points to a low glycolytic rate as well as ATP and NADH yield. However, while most of the accumulated amino acids might not result from reactions of the glycolytic pathway or TCA-cycle intermediates, it might be due to the inhibition or depletion of protein biosynthesis. The accumulation of amino acids and sugars, well-known for their osmoprotectant role (Dias et al., 2015), might be an indicative that *B. boehmii* and *C. mopane* have a stress memory that supports survival under fire conditions or allows crosstalk between different environmental stimuli related to fire [e.g., high temperature (30–40°C); low rainfall (NNR: 600–1400 mm; LNP: 360–500 mm); low fertile soil (NNR: sandy; LNP: limestone and rocky)], leading to a transcriptional/somatic stress memory (phenotypic plasticity) (Bradshaw et al., 2011; Ding et al., 2012; Feng et al., 2016). In fact, thermo-tolerance in plants related to heat-stress memory is actively maintained due to the temporary activation of heat-inducible genes (memory genes) after a priming heat stress (Charng et al., 2006; Feng et al., 2016).

The different primary metabolite profile between *B. boehmii* and *C. mopane* suggests distinct response mechanisms to fire. On one hand, the accumulation of sugars and amino acids in *B. boehmii* is probably related to osmoprotection, “scavenging” of ROS and pH regulation. On the other hand, the accumulation of amino acids and reduction of sugars in *C. mopane* is probably related to osmoprotection processes and energy production, respectively.

The differences between the primary metabolites profiles of *C. mopane* plants from the two sites of LNP might be related to intraspecific genetic polymorphism or to ecologic factors, such as geomorphologic conditions and composition of woody vegetation. Indeed, the soil type determines the composition of woody vegetation of LNP. While in the rocky soils of Libombo north, *Combretum apiculatum* (Sond) was the only sub-dominant woody species observed, in calcareous soils from Calcrete, a variety of sub-dominant woody species, such as *Eucleia* ssp, *Grewia* spp, *Dalbergia melanoxylon* (Guill & Perr) occur, suggesting that the dominant mopane plants from Calcrete are subjected to a higher competition with woody vegetation than mopane plants from Libombo north (Stalmans et al., 2004; Govender et al., 2015). Overall, these factors might influence the response of *C. mopane* to stress, thus explaining the differences in the metabolite profiles.

In summary, the primary metabolite profiles of *B. boehmii* and *C. mopane* are different, except for the amino acids Ala, Ser, Thr, Phe, GABA, glutamate (Glu), Gly, Ile, Pro, tyrosine (Tyr) and Val, which probably represent a convergent response of both species to fire. That convergent response may reflect the fact that both species belong to the same family and probably share common genes with the same expression pattern and regulation of metabolic pathways of amino acid metabolism in environmental stress conditions.

The use of a well-established GC-TOF-MS metabolite profiling approach to measure known primary metabolites (e.g., sugars, amino and organic acids) has proven to be a powerful analytical tool to improve our understanding of how and to which extent *B. boehmii* and *C. mopane* metabolism readjusts to fire exposure in the Miombo and Mopane ecosystems, respectively. The observed accumulation of amino acids and sugars in *B. boehmii* and *C. mopane* might be related to fire tolerance mechanisms in these species, such as osmoprotection, “scavenging” of ROS, and high rates of energy production.

This study represents a pioneering approach to profile the primary metabolome of *B. boehmii* and *C. mopane*, and constitutes an important starting point to further investigate biochemical markers of thermal stress tolerance in other African leguminous species from the Miombo and Mopane ecosystems. To complement this study, the characterization of the secondary metabolome of *B. boehmii* and *C. mopane* with LC-MS/MS approaches is being carried out. Preliminary results indicate that these plants contain added-value phenolic compounds with antibacterial and antioxidant properties, which levels have shown to increase with fire frequency.

## Author Contributions

NR, AR-B, and CA conceived and designed the project. IM collected the samples. JD and TJ were responsible for all plant metabolomics workflows. JD, TJ, and CA analyzed the data. CA and AR-B contributed with reagents, materials, and analysis tools. JD, TJ, IM, AR-B, and CA wrote the paper.

## Conflict of Interest Statement

The authors declare that the research was conducted in the absence of any commercial or financial relationships that could be construed as a potential conflict of interest.
